# Publisher Correction: Propagation of hippocampal ripples to the neocortex by way of a subiculum-retrosplenial pathway

**DOI:** 10.1038/s41467-020-17080-0

**Published:** 2020-07-07

**Authors:** Noam Nitzan, Sam McKenzie, Prateep Beed, Daniel Fine English, Silvia Oldani, John J. Tukker, György Buzsáki, Dietmar Schmitz

**Affiliations:** 1 Charité-Universitätsmedizin Berlin, Corporate member of Freie Universität Berlin, Humboldt-Universität zu Berlin, and Berlin Institute of Health, Neuroscience Research Center, Berlin, Germany; 2 Neuroscience Institute and Department of Neurology New York University Langone Medical Center, New York, NY 10016 USA; 3 Center for Neurodegenerative Diseases (DZNE), Berlin, Germany; 4 Center for Neural Science, New York University, New York, NY 10016 USA; 5 Cluster of Excellence NeuroCure, Berlin, Germany; 6 Einstein Center for Neurosciences, Berlin, Germany; 7 Present Address: School of Neuroscience, College of Science, Virginia Tech, VA 24061 USA

**Keywords:** Hippocampus, Neural circuits

Correction to: *Nature Communications* 10.1038/s41467-020-15787-8, published online 23 April 2020.

The original version of this Article contained an error in Fig. 5 that was inadvertently introduced during production. The black line depicting the confidence interval was shifted in Fig. 5e, first row, right panel. This error has been corrected in both the PDF and HTML versions of the Article.

