# Supplementary material for: Propagation of hippocampal ripples to the neocortex by way of a subiculum-retrosplenial pathway
Source: Nat Commun. 2020 Apr 23;11:1947. doi: 10.1038/s41467-020-15787-8 (PMC7181800; doi:10.1038/s41467-020-15787-8)
Supplement: Supplementary file 3 — Reporting Summary [file 41467_2020_15787_MOESM3_ESM.pdf]

nature research

Corresponding author(s): Dietmar Schmitz

Last updated by author(s): Mar 20, 2020

Reporting Summary

Nature Research wishes to improve the reproducibility of the work that we publish. This form provides structure for consistency and transparency in reporting. For further information on Nature Research policies, see Authors & Referees and the Editorial Policy Checklist .

Statistics

For all statistical analyses, confirm that the following items are present in the figure legend, table legend, main text, or Methods section.

n/a	Confirmed	
☐	☒	The exact sample size (*n*) for each experimental group/condition, given as a discrete number and unit of measurement
☐	☒	A statement on whether measurements were taken from distinct samples or whether the same sample was measured repeatedly
☐	☒	The statistical test(s) used AND whether they are one- or two-sided<br>*Only common tests should be described solely by name; describe more complex techniques in the Methods section.*
☐	☒	A description of all covariates tested
☐	☒	A description of any assumptions or corrections, such as tests of normality and adjustment for multiple comparisons
☐	☒	A full description of the statistical parameters including central tendency (e.g. means) or other basic estimates (e.g. regression coefficient) AND variation (e.g. standard deviation) or associated estimates of uncertainty (e.g. confidence intervals)
☐	☒	For null hypothesis testing, the test statistic (e.g. *F*, *t*, *r*) with confidence intervals, effect sizes, degrees of freedom and *P* value noted<br>*Give P values as exact values whenever suitable.*
☒	☐	For Bayesian analysis, information on the choice of priors and Markov chain Monte Carlo settings
☒	☐	For hierarchical and complex designs, identification of the appropriate level for tests and full reporting of outcomes
☒	☐	Estimates of effect sizes (e.g. Cohen's *d*, Pearson's *r*), indicating how they were calculated

Our web collection on statistics for biologists contains articles on many of the points above.

Software and code

Policy information about availability of computer code

Data collection	In-vitro data were collected using Igor Pro; In-vivo data were collected using Intan RHD2000.
Data analysis	Data were analyzed using MATLAB (2018a, license number 40638465) with the majority of the code adapted from https://github.com/buzsakilab/buzcode. Spike sorting was performed using Kilosort (https://github.com/cortex-lab/KiloSort/). Spike sorted data were manually curated using Klusters and LFP data were visualized using Neuroscope (http://neurosuite.sourceforge.net/). ICA was performed using EEGLAB toolbox.

For manuscripts utilizing custom algorithms or software that are central to the research but not yet described in published literature, software must be made available to editors/reviewers. We strongly encourage code deposition in a community repository (e.g. GitHub). See the Nature Research guidelines for submitting code & software for further information.

Data

Policy information about availability of data

All manuscripts must include a data availability statement. This statement should provide the following information, where applicable:

- Accession codes, unique identifiers, or web links for publicly available datasets
- A list of figures that have associated raw data
- A description of any restrictions on data availability

Parts of the data have been deposited on https://buzsakilab.nyumc.org/datasets/. The remaining data will be made available upon request.

Field-specific reporting

Please select the one below that is the best fit for your research. If you are not sure, read the appropriate sections before making your selection.

Life sciences study design

All studies must disclose on these points even when the disclosure is negative.

Sample size	No statistical methods were used to predetermine sample sizes. Sample sizes were chosen to be similar to those reported in previous publications using extracellular silicon probe recordings and in-vitro patch clamp recordings (Stark et al., Neuron 2014; Justus et al., Nature Neuroscience 2017).
Data exclusions	For in-vitro experiments, we used pre-established criteria of systematically excluding cells where access resistance fluctuated by more than 20% (Maier et al., 2011).
Replication	All findings reported in this study were replicable across individual subjects and across sessions. All experiments were repeated across at least 3 different subjects. Successful identification of gRSC ripples was contingent upon histologically confirmed targeting of superficial gRSC layers.
Randomization	Samples were allocated to groups based on animal strain (i.e. Wild-type or transgenic). Randomization was not relevant for this study as wild-type and transgenic animals were not directly compared.
Blinding	Experiments and analyses were not performed blind to group identity (i.e. animal strain). In experiments involving optogenetic inhibition, ripple detection was performed blindly to light stimulation. Bliding was not relevant for this study as wild-types and transgenic animals were not compared againt each other.

Reporting for specific materials, systems and methods

We require information from authors about some types of materials, experimental systems and methods used in many studies. Here, indicate whether each material, system or method listed is relevant to your study. If you are not sure if a list item applies to your research, read the appropriate section before selecting a response.

Materials & experimental systems

n/a	Involved in the study
☐	☒ Antibodies
☒	☐ Eukaryotic cell lines
☒	☐ Palaeontology
☐	☒ Animals and other organisms
☒	☐ Human research participants
☒	☐ Clinical data

Methods

n/a	Involved in the study
☒	☐ ChIP-seq
☒	☐ Flow cytometry
☒	☐ MRI-based neuroimaging

Antibodies

Antibodies used	GAD-67 primary mouse antibody (diluted 1:500, MAB5406, Millipore)<br>Secondary anti-mouse Alexa 555 (1:500, A-21424, Invitrogen)<br>Streptavidin conjugated to Alexa 647 (1:500, S32357, Invitrogen)
Validation	Antibodies used in this study where previously used, and validated, in a study from the same lab (Winterer et al., Cell Reports, 2019).

Animals and other organisms

Policy information about studies involving animals; ARRIVE guidelines recommended for reporting animal research

Laboratory animals	All data used for this study were collected from both male and female mice, 2-3 months old, of the following mouse lines:<br>8 x C57BL/6 mice (JAX: 005304)<br>24 x VGlut2-Cre mice (JAX: 016963)<br>4 x CaMKII-Cre::Ai32 mice (JAX # 005359 with JAX # 012569)<br>Animals were housed in a 12/12 light-dark cycle (ambient temperature: 21°C; humidity: 54%).
Wild animals	The study did not involve wild animals
Field-collected samples	The study did not involve samples collected from the field
Ethics oversight	All experiments were conducted in accordance with European guidelines and with permission from local regulatory authorities (Berlin Landesamt für Gesundheit und Soziales, permits G0092/15 and G0150/17 and the Institutional Animal Care and Use Committee of New York University Medical Center).

Note that full information on the approval of the study protocol must also be provided in the manuscript.
